# Oropharyngeal Manifestations in Patients with HIV from Northeastern Romania

**DOI:** 10.3390/medicina61050855

**Published:** 2025-05-06

**Authors:** Amelia Elena Surdu, Isabela Ioana Loghin, Victor Daniel Dorobăţ, Vlad Hârtie, Șerban Alin Rusu, Ion Cecan, Amelia Andreea Mihăescu, Otilia Eva, Carmen Mihaela Dorobăț

**Affiliations:** 1 Department of Implantology, Removable Prostheses and Technology, Faculty of Dental Medicine, “Grigore T. Popa” University of Medicine and Pharmacy, 700115 Iasi, Romania; amelia.surdu@dentesse.ro; 2 Department of Infectious Diseases, Faculty of Medicine, “Grigore T. Popa” University of Medicine and Pharmacy, 700115 Iasi, Romania; 3 Department of Infectious Diseases, “St. Parascheva” Clinical Hospital of Infectious Diseases, 700116 Iasi, Romania; rususerbanalin@yahoo.com (Ș.A.R.); ion11cecan@gmail.com (I.C.); ameliamihaescu@gmail.com (A.A.M.); evaotilia@yahoomail.com (O.E.); carmendorobat@yahoo.com (C.M.D.); 4 Department of Intensive Care, University Hospital of Emergency, 050098 Bucharest, Romania; victordorobat@yahoo.com; 5 Department of Intensive Care, Clinical Hospital of Emergency “Prof. Dr. Nicolae Oblu”, 700309 Iasi, Romania; vladhartie@yahoo.com; 6 Department of Otolaryngology, Faculty of Medicine, Apollonia University, 700511 Iasi, Romania

**Keywords:** stomatognathic system, ENT manifestations, dental diagnosis, HIV infection, oral disease

## Abstract

*Backgrounds and objective*: Disorders in the stomatognathic system and otorhinolaryngologic manifestations are frequently observed in individuals living with HIV. Ear, neck, and throat (ENT) signs and symptoms often serve as critical markers of treatment failure, particularly in the advanced stages of HIV infection. This article aims to evaluate and consolidate recent developments in the treatment and management of otorhinolaryngological manifestations in HIV-positive patients. *Materials and methods*: We carried out a retrospective clinical investigation of patients admitted with HIV/AIDS in the northeastern region of Romania, hospitalized in the “St. Parascheva” Clinical Hospital of Infectious Diseases in Iasi. We followed the viro-immunological status correlated with patients’ otolaryngology and dental symptomatology, aiming to emphasize the comorbidities of HIV/AIDS cases. The study period spanned from 1 January 2020 to 30 November 2024. *Results*: There were a total of 552 recorded cases of oropharyngeal manifestations in patients with HIV. They were more frequent in men (358 cases, 64.85%) than women (194 cases, 35.15%). The majority of cases were young adults, aged 30 to 39 years, comprising 255 patients (46.19%), and most cases (36.85%) had CD4+ T-lymphocyte values between 200 and 499 cells/μL. The most frequent diagnosis was oral candidiasis, recorded in 335 male and 174 female cases (509, 92.21% total). Other notable conditions included gingivitis/periodontitis, sinusitis/rhinosinusitis, mastoiditis, and dental abscesses, albeit at lower frequencies. Notably, antifungal therapy with fluconazole was the most frequently employed treatment, followed by aminopenicillins and fluoroquinolones. With respect to the antiretroviral treatment, 83.69% of cases were prescribed a single-pill regimen. *Conclusions*: The key to the management of HIV-positive patients is a multidisciplinary approach, including an ENT specialist and access to antiretroviral therapy.

## 1. Introduction

Human immunodeficiency virus (HIV) and acquired immunodeficiency syndrome (AIDS) have emerged as a global pandemic, affecting approximately 40 million individuals worldwide. This widespread health crisis underscores the critical need for effective prevention, diagnosis, and treatment strategies to mitigate its impact on public health and reduce the global disease burden [1].

Human immunodeficiency virus (HIV) is a persistent viral infection that progressively undermines the immune system by selectively infecting and depleting CD4+ T-lymphocytes, which are crucial in orchestrating immune responses. If left untreated, HIV can advance to acquired immunodeficiency syndrome (AIDS), a stage marked by profound immunosuppression and increased susceptibility to opportunistic infections and various malignancies. HIV primarily disseminates to lymphoid tissues through the bloodstream after crossing mucosal barriers. The progression from HIV to AIDS is typically characterized by a significant decline in the CD4+ T cell count, accompanied by the emergence of clinical conditions that are rare in individuals with intact immune systems [1,2,3].

According to the World Health Organization (WHO), HIV remains a major global health concern, ranking as the ninth leading cause of mortality in low-income countries and the sixth leading cause of death among communicable diseases. Despite its significant burden, there has been substantial progress in reducing HIV-related mortality. Between 2000 and 2019, HIV-related deaths decreased by 59%, reflecting advancements in prevention, diagnosis, and treatment. Furthermore, scaling up the AIDS response in low- and middle-income countries could prevent an estimated 28 million new HIV infections and 21 million AIDS-related deaths between 2015 and 2030, underscoring the critical need for sustained efforts to combat the epidemic [4].

Since the introduction of antiretroviral therapy (ART), the prognosis for individuals living with HIV has significantly improved, transforming the condition from a fatal illness into a manageable chronic disease for many. ART effectively suppresses viral replication and prevents the progression of AIDS. However, despite advancements in viral control, HIV-positive individuals remain at an elevated risk for various non-AIDS-defining conditions, such as cardiovascular diseases, neurocognitive impairments, and malignancies. This continued risk is attributed to persistent immune activation and chronic inflammation, which are hallmarks of HIV infection even in individuals with well-controlled viral loads [5].

Disorders in the stomatognathic system and otorhinolaryngologic manifestations are frequently observed in individuals living with HIV. A thorough understanding of the disease’s progression is crucial for accurate diagnosis and effective management. Ear, neck, and throat (ENT) signs and symptoms often serve as critical markers of treatment failure, particularly in the advanced stages of HIV infection [6].

Oropharyngeal manifestations, as part of opportunistic infections, are well recognized in individuals living with HIV and play a significant role in the clinical staging of the disease. These manifestations, which include infections of the oral cavity, sinuses, throat, and ears, are indicative of immune system deterioration and are commonly observed as HIV progresses. The World Health Organization includes these opportunistic infections in its clinical staging system, highlighting their importance in monitoring the progression of HIV and guiding treatment decisions. These manifestations often signal a compromised immune response, necessitating early intervention to prevent further complications and improve patient outcomes [6,7].

Oral lesions associated with HIV infection may result from fungal, viral, or bacterial pathogens, as well as neoplastic or idiopathic processes. These lesions are indicative of the immunosuppressed state induced by HIV, rendering patients more susceptible to severe presentations of conventional periodontal conditions. This increased severity underscores the need for vigilant oral health monitoring in this population to facilitate the timely diagnosis and management of underlying systemic and opportunistic infections [8].

Romania is home to one of the largest pediatric HIV cohorts in Europe, largely resulting from nosocomial transmission events during the late 1980s and early 1990s. A significant proportion of these patients were infected with the F1 HIV-1 subtype, a genetic variant predominantly found in Romania, which continues to impact long-term clinical outcomes. Additionally, disparities in healthcare access between urban and rural populations may delay the diagnosis and treatment of HIV-related complications. This unique combination of historical, virological, and socioeconomic factors underscores the clinical importance of this regional dataset [9].

This study aims to evaluate and consolidate recent developments in the treatment and management of otorhinolaryngological manifestations in HIV-positive patients. In the context of pathological changes in the stomatognathic system observed in patients with HIV/AIDS, it is essential to have a thorough understanding of these pathologies, allowing other specialties to respond appropriately and refer patients promptly to HIV/AIDS departments. We hypothesize that lower CD4+ T cell counts, smoking status, rural residence, and periodontal disease are significantly associated with the presence of oral candidiasis in this population.

## 2. Materials and Methods

### 2.1. Study Design and Database Information

To highlight the characteristics and ENT-related comorbidities of HIV/AIDS cases, we conducted a retrospective clinical investigation of patients with HIV/AIDS in the northeastern region of Romania who were admitted to the Clinical Hospital of Infectious Diseases in Iasi, based on hospital medical data. The study period spanned from 1 January 2020 to 30 November 2024.

Patients over the age of 18 who were hospitalized during the study period at our Regional HIV/AIDS Center in northeastern Romania were included in this study if they tested positive for HIV via an enzyme-linked immunosorbent assay (ELISA) and had their diagnosis confirmed via Western blot (WB). HIV plasma viral loads and CD4+ T cell counts were measured for all confirmed cases. The serological testing process involved two ELISA tests for initial screening, followed by a confirmatory Western blot performed by the regional public health management network’s epidemiologists. Following confirmation, patients were referred to the local HIV/AIDS facility for comprehensive evaluation and management.

### 2.2. Ethical Approval

The “St. Parascheva” Clinical Hospital of Infectious Diseases in Iasi, Romania, cleared this study (Febr. 2025; Approval No. 3/12 February 2025). At admission, every individual signed documentation indicating informed consent.

### 2.3. Study Variables

Demographic data, stratified by age and sex, were systematically documented alongside detailed anamnesis and clinical characteristics. The dataset encompassed extensive viro-immunological assessments, including serological testing for opportunistic pathogens such as *Toxoplasma gondii*, *Cytomegalovirus*, hepatitis B and C viruses, and *Candida* spp. infections. HIV infection stages were determined following established immunological and clinical criteria, while the initiation of antiretroviral therapy (ART) and the longitudinal progression of the disease were meticulously recorded to ensure comprehensive patient profiling and outcome analysis. We also focused on viro-immunological evaluation to monitor the status of immunosuppression.

To ensure consistent treatment outcomes, all patients were routinely assessed by an ENT specialist and a dentist, addressing the high prevalence of ENT and stomatognathic complications in this population. Clinical examination—tongue scraping, followed by microscopic examination and fungal cultures (for oral candidiasis)—and imagistic evaluation (sinus X-ray) were used to confirm the diagnosis. The collected data, encompassing both retrospective and current clinical findings, were analyzed to identify trends and correlations in disease progression. In one case, we used a histological tissue biopsy for the diagnosis.

Of the patients admitted to our hospital, some were not included in this study based on the following exclusion criteria: positive viro-immunological status, adherence to treatment, and absence of symptoms in the ENT and dental fields. Patients were also excluded from this study if they had a CD4+ T-lymphocyte count greater than 200 cells/mm^3^; an HIV viral load below 40 copies/mL, or an undetectable load; and no ENT or stomatognathic manifestations at the time of evaluation. These criteria were applied to ensure that the study population consisted of individuals with clinically significant immunosuppression who were at risk for HIV-related ENT and oral complications. This approach allowed for a more focused analysis of predictors associated with these manifestations.

The FACS (fluorescence-activated cell sorting) count technique was used to measure and quantify cell subpopulations, particularly CD4+ and CD8+ T-lymphocytes in the blood. A FACS count analyzer uses flow cytometry, where cells pass through a laser beam. Based on fluorescent cellular markers (fluorescently labeled monoclonal antibodies), the device detects and counts different types of cells. It relies on monoclonal antibodies tagged with fluorochromes that specifically bind to CD4 or CD8 molecules on the cell surface. Normal LyT CD4 values were recorded between 500 cells/mm^3^ and 1400 cells/mm^3^.

Cepheid’s GeneXpert^®^ system (Sunnyvale, CA, USA) was used for HIV viral load testing. It utilizes real-time polymerase chain reaction (PCR) technology for the rapid and accurate detection of HIV RNA, making it a crucial tool for monitoring viremia in HIV patients. The GeneXpert system amplifies and detects HIV RNA using a cartridge-based PCR assay. A viral load below 40 copies/mL was deemed undetectable; a load above 40 copies/mL was deemed detectable.

The Centers for Disease Control and Prevention (CDC), located in Atlanta, states that the specific CD4+ T-lymphocyte count or the CD4+ T-lymphocyte percentage of the total T-lymphocyte cells is used to define the HIV infection stage. There are three phases of HIV infection and AIDS: stage 1 occurs when the CD4+ T-lymphocyte counts exceed 500 cells/μL, stage 2 occurs when they fall between 200 and 499 cells/μL, and stage 3 occurs when they fall below 200 cells/μL. Stages 1 and 2 signify HIV infection, and stage 3 denotes AIDS.

### 2.4. Study Setting

The “St. Parascheva” Clinical Hospital of Infectious Diseases in Iasi serves as the main referral hospital for the Moldova area of Romania. There are six pavilions within it. An Infectious Diseases and HIV/AIDS Regional Center is located in Pavilion V, where patients are routinely assessed following CDC and EACS guidelines.

All blood tests were performed at the hospital’s central laboratory, and the molecular biology lab was used to assess the patients’ HIV plasma viral loads and CD4+ T cell counts. Cepheid’s GeneXpert^®^ system was used in conjunction with RT-PCR for HIV-1 to measure viral load levels and assess HIV viremia. Viral loads were considered undetectable if they were below 40 copies/mL, and detectable otherwise.

The Regional HIV/AIDS Center in Iasi maintains comprehensive medical records for its 1724 active patients, facilitating continuous care and monitoring. Each patient’s medical file includes detailed information about their immunological and virological status, comorbid conditions, and adherence to ART regimens.

### 2.5. Statistical Analysis

The correlation between the results, clinical data, and demographic variables was assessed using a Pearson test in the XLSTAT program, version 2019, and the correlation coefficients of Kendall’s Tau were determined. Statistical analysis was performed using Statistical Software for Excel (XLSTAT), version 2019.4.

## 3. Results

The active records of the Regional HIV/AIDS Center from Iasi covered 1724 patients. From 1st January 2020 to 31st November 2024, among the patients admitted at the Iasi Regional HIV/AIDS Center who visit periodically for viro-immunological evaluation, 552 were selected based on their presentation of acute lesions of the stomatognathic and otorhinolaryngologic system. Admissions were lowest in 2020, with only 281 admissions and 3 cases (1.1%) with stomatognathic lesions, due to the COVID-19 pandemic. The highest number of admissions was recorded in 2024, with 1239 cases, including 165 involving stomatognathic lesions (Table 1).

Young adults made up the majority of cases, with 255 patients (46.19%) between the ages of 30 and 39, followed by patients in the 40–49 age range (23.91%), those in the 50–59 age group (10.86%), those in the 18–29 age group (10.68%), and those over 60 years old (41 patients, 7.42%) (Table 1). The study group’s average age was 37.31 years old. Men were more likely to be infected (358 cases, 64.85%) than women (194 cases, 35.15%) (Table 2). The remaining 359 cases (65%) were from rural areas, while 193 patients (35%) were from northeastern Romania’s urban area.

Considering the route of transmission, all cases reported a possible cause. The most common was the sexual transmission route, with a total of 529 cases (95.83). Perinatal transmission was recorded in 17 cases (3.07%), and intravenous drug use was recorded in 6 cases (1.08%). Young adult males (ages 21 to 50) were the most afflicted group in terms of the sexual route of transmission (heterosexual men and MSM—men who have sex with men—accounting for 36.21% of cases) (Table 3).

The study group from the Iasi HIV/AIDS Regional Center was virologically and immunologically evaluated.

**Figure 1:**
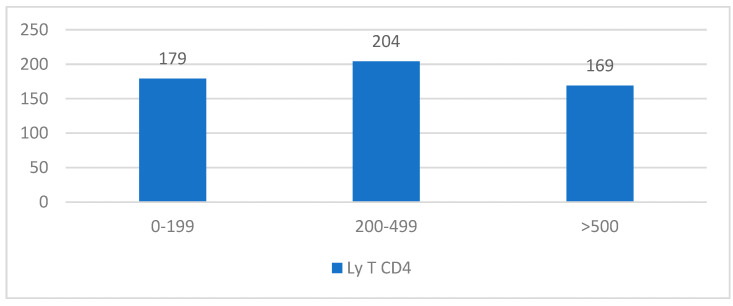
Distribution of cases by CD4+ T-lymphocyte level (cells/μL).

The average CD4+ T-lymphocyte level was 372.34 cells/μL: 32.52% of cases had levels between 1 and 199 cells/μL, 36.85% of cases had values between 200 and 499 cells/μL, and 30.63% had values exceeding 500 cells/μL (Table 4, Figure 1). Males were primarily affected, exhibiting a decreased total CD4+ T-lymphocyte level, according to the gender–lymphocyte correlation. According to Table 4 and Figure 1, the average HIV viral load was 429.271 copies/m.

The study group was screened for the most prevalent co-infections linked to HIV/AIDS. The findings demonstrate that various opportunistic infections were present in over half (96.04%) of the patients admitted to our clinic during the study period.

The results show that the most frequent co-infections were oral candidiasis (92.21% of patients); hepatitis B (19.91%); tuberculosis, either recent-onset or recorded in past admissions (14.84%); and hepatitis C (4.88%). Males were more affected than females, exhibiting a higher percentage of co-infections (Table 5).

The ENT manifestations observed in this study, with oral candidiasis being the most frequent diagnosis, were recorded in 335 male and 174 female cases (509, 92.21% total). Sinusitis/rhinosinusitis was the second most common condition, affecting 9 females and 8 males, with an additional case associated with temporomandibular arthritis in a female patient (18 cases, 3.26% total). Ethmoiditis was noted in four females (0.72%) and two males (0.36%), while dental abscesses and rhinosinusitis occurred in five male patients (0.90%). Mastoiditis was equally distributed, with three cases each in males and females (six cases, 1.08% total). Nasal septum deviation was reported in one female, and in two males in combination with sinusitis/rhinosinusitis (three cases, 0.54%). Otitis occurred exclusively in males (two cases, 0.36%), and temporomandibular arthritis was observed in one female (0.18%). This distribution highlights the predominance of oral candidiasis and sinusitis/rhinosinusitis, with certain conditions exhibiting gender-specific trends. In a few cases, dental abscesses were also diagnosed. Gingivitis and periodontitis were found in 552 patients with HIV. Gingivitis was diagnosed in 32% (n = 177) of patients, while periodontitis was observed in 36% (n = 199). The high prevalence of periodontal diseases in this cohort suggests a significant burden of oral inflammatory conditions, which may be exacerbated by immunosuppression, poor oral hygiene, and smoking (Table 6). One case recorded upper lip verrucous carcinoma, diagnosed via tissue biopsy.

Multivariable regression analysis was used to evaluate the predictors of oral candidiasis in HIV-positive patients. The dependent variable was the presence of oral candidiasis, while the independent variables included the CD4+ T-lymphocyte count, HIV viral load, gender, ART adherence, co-infections (HBV, HCV, and tuberculosis), sociodemographic characteristics, smoking status, and periodontal conditions (gingivitis and periodontitis) (Table 7).

The CD4+ T-lymphocyte count emerged as the most significant predictor, with a coefficient of −0.30 (*p* < 0.001), indicating a 26% reduction in candidiasis risk per 100-cell/µL increase (OR = 0.74). Among patients with CD4+ counts below 200 cells/µL, the prevalence of candidiasis exceeded 90%, confirming the role of severe immunodeficiency as a major risk factor.

Smoking was another highly significant predictor, with a coefficient of +1.25 (*p* < 0.001), corresponding to a 3.5-fold increased risk of candidiasis among smokers compared to non-smokers. Given that 60% of smokers in this study were diagnosed with *Candida* spp., this finding underscores the role of tobacco use in promoting fungal colonization and infection.

Sociodemographic characteristics (urban vs. rural) also played a significant role (B = −1.36, *p* < 0.001, OR = 0.26), indicating that patients from rural areas had a fourfold increased risk of developing candidiasis compared to urban residents. Notably, 65% of the study population resided in rural areas, further reinforcing healthcare access disparities as a critical determinant of opportunistic infections.

In terms of periodontal conditions, gingivitis (B = +0.85, *p* = 0.004, OR = 2.35) and periodontitis (B = +1.10, *p* = 0.002, OR = 3.02) were significant predictors, demonstrating that patients with these conditions were more likely to develop oral candidiasis. In this cohort, 32% of patients had gingivitis, while 36% had periodontitis, suggesting that periodontal disease is prevalent in HIV-positive individuals and contributes to higher candidiasis rates.

In contrast, HIV viral load, gender, ART adherence, and co-infections (HBV, HCV, and tuberculosis) were not statistically significant predictors (*p* > 0.15), suggesting that their impact on candidiasis is mediated primarily through CD4+ depletion rather than direct effects. Despite this, patients with detectable HIV viremia had higher overall candidiasis rates.

*Candida* spp. diagnosis among smokers and non-smokers was based on lingual swab results from patients with HIV/AIDS in our cohort. Among 442 smokers (80% of patients), 60% (n = 265) were diagnosed with *Candida* spp., indicating a strong association between smoking and fungal colonization. In contrast, among 110 non-smokers (20% of patients), only 20% (n = 22) tested positive for *Candida* spp. after lingual swab (Table 8).

For every patient diagnosed with an oropharyngeal manifestation and dental abscesses, empirical antibiotic/antifungal treatment was initiated with favorable outcomes. Aminopenicillins, specifically amoxicillin–clavulanate, were used in 2.35% of patients for 7–10 days. Second-generation cephalosporins, represented by cefuroxime, and third-generation cephalosporins, such as ceftriaxone and ceftazidime, were administered to 0.90% and 1.44% of patients, respectively, for a treatment duration of 7 days. Fluoroquinolones, particularly ciprofloxacin, were used in 2.17% of cases, with treatment lengths ranging from 7 to 14 days. Notably, antifungal therapy with fluconazole was the most frequently employed treatment, accounting for 92.21% of patients, with a standard duration of 3 days (Table 9).

The patients diagnosed with *Candida* spp. infection received fluconazole 150 mg per day as the primary antifungal therapy. At the follow-up assessment, 80% (n = 407) of treated patients had a negative tongue swab for *Candida* spp., indicating successful resolution of the infection. The remaining 20% (n = 102) of patients exhibited persistent colonization or infection, which may suggest fluconazole resistance, suboptimal adherence, or severe immunosuppression (Table 10).

Antiretroviral therapy was prescribed for every patient identified at the Iasi HIV/AIDS Regional Center. Consequently, a single-pill regimen was administered in 83.69% of instances, while a regimen that considered their comorbidities and adhered to guidance from www.hiv-druginteractions.org accessed on 20 March 2025, was prescribed to the remaining 16.31% of cases (Table 11).

Integrase inhibitor-based antiretroviral regimens were the most commonly used (309 patients, 55.97%), followed by nucleoside reverse transcriptase inhibitor (NRTI)-based regimens with non-nucleoside reverse transcriptase inhibitors (NNRTIs), which were used in 200 patients (36.23%), and protease inhibitor-based regimens (29 patients, 5.25%). Fourteen patients (2.53%) were treated with other antiretroviral regimens, such as CCR5 inhibitors, NRTIs + integrase inhibitors, or protease inhibitors + integrase inhibitors (Table 12).

## 4. Discussion

This study highlights the significant burden of oropharyngeal manifestations among people living with HIV in northeastern Romania, emphasizing their correlation with advanced stages of immunosuppression. Oral candidiasis emerged as the most prevalent condition, affecting 92.21% of patients, reflecting its role as a primary marker of disease progression. Other notable conditions included gingivitis/periodontitis, sinusitis/rhinosinusitis, mastoiditis, and dental abscesses, albeit at lower frequencies. Gender differences were evident, with males exhibiting higher rates of co-infections and oropharyngeal complications.

Oral lesions, often accompanied by nonspecific symptoms, are among the earliest clinical manifestations of HIV infection and serve as critical markers for disease progression to AIDS. These lesions are reported in approximately 30% of individuals with HIV and up to 80% of patients diagnosed with AIDS, highlighting their prevalence and diagnostic significance in the context of immunosuppression and disease advancement [10,11,12]. Among the patients admitted, 552 patients (14.19%) were selected based on their reported presentation of acute lesions of the stomatognathic and otorhinolaryngologic systems.

The Oral Manifestations of Human Immunodeficiency Virus, as classified by the EC–Clearinghouse and the World Health Organization (WHO), categorize oral lesions based on their relationship with HIV infection into three groups: those strongly associated with HIV, those less commonly associated, and those observed in individuals with HIV [13]. We observed a predominance of oral candidiasis and gingivitis/periodontitis, with certain conditions exhibiting gender-specific trends. In a few cases, dental abscesses were also diagnosed.

In alignment with the EC–Clearinghouse–WHO classification, common oral manifestations in HIV/AIDS patients, such as candidiasis, oral hairy leukoplakia, periodontal disease, and Kaposi sarcoma, continue to be frequently reported. Additionally, conditions such as oral mucosal hyperpigmentation, HIV-related salivary gland disorders, and potentially HIV-associated oral ulcerations are increasingly recognized, suggesting that improved access to antiretroviral therapy (ART) in recent years has influenced the prevalence and presentation of these manifestations. Furthermore, variations in the frequency of HIV-related oral conditions between developed and developing regions are evident, likely reflecting disparities in socioeconomic, cultural, and healthcare accessibility factors [13,14,15,16]. Our findings support these observations, as oral candidiasis was the most common manifestation in the study group.

In their study, Dongo M. et al. observed that in a total of 750 men and 237 women, oral manifestations were less prevalent in females than in males [17,18]. Similarly, we recorded that stomatognathic system oral lesions were more frequent in men (358 cases, 64.85%) than in women (194 cases, 35.15%).

Sex-based differences appear to influence the prevalence of oral manifestations associated with HIV/AIDS. Research indicates that oral hairy leukoplakia and Kaposi sarcoma are more commonly observed in men, whereas conditions such as linear gingival erythema and aphthous ulcers are more frequently reported in women. These findings highlight potential variations in clinical presentation that may be influenced by biological or environmental factors, emphasizing the need for personalized approaches to diagnosis and management [19,20,21]. We did not, however, record any cases of oral hairy leukoplakia or Kaposi sarcoma.

Candidiasis is the most prevalent opportunistic fungal infection in HIV-positive individuals, with a prevalence of 17–75%, primarily caused by *Candida albicans*, but also involving other species like *C. glabrata* and *C. tropicalis*. Oral candidiasis is commonly one of the first clinical manifestations observed during the acute phase of HIV infection, indicating early immune system involvement. It is a significant indicator of immune system compromise and can precede a formal HIV diagnosis [10,13]. Its prevalence exhibits significant variability across different studies. In agreement with previous findings, oral candidiasis was observed in 92.21% of the cases in our study.

Key risk factors include severe immunosuppression (CD4+ T cell count <200 cells/μL), tobacco use, broad-spectrum antibiotics, corticosteroids, and local contributors such as partial dentures, reduced saliva flow, and nutritional deficiencies. Clinically, it presents as pseudomembranous candidiasis, erythematous candidiasis, or angular cheilitis, necessitating prompt diagnosis and management to prevent complications in immunocompromised individuals [22,23]. In our study, oral candidiasis was the most frequent diagnosis; in some cases, it was associated with low immunological status.

Based on the disease topography in 552 patients, Keita A. et al. found rhinitis (80.2%), rhinosinusitis (18.9%), and epistaxis (0.9%) to be present in 55.7% of cases with ENT involvement. A total of 50.9% of cases involved the oral cavity and oropharynx, including tonsillitis (37.2%), oral thrush (3.8%), and candidiasis (54.3%) [24]. Our study found that oral candidiasis was the most frequent diagnosis, recorded in 335 male and 174 female cases (509, 92.21% total). Gingivitis was the second most common condition, affecting 374 cases (67.75%).

Variations in the prevalence of oral lesions among different anatomical sites appear to correlate with educational level, with regions such as the floor of the mouth and buccal mucosa being more frequently affected in individuals with informal education. This disparity may reflect differences in oral hygiene practices. Previous studies, such as those by Johnson and Tirwomwe et al., suggest that the increased frequency of oral lesions in patients with HIV/AIDS is linked to poor dietary and oral hygiene habits, discomfort during tooth brushing, and difficulties with chewing or swallowing. These observations underscore the influence of educational and behavioral factors on the manifestation of oral conditions in HIV-positive individuals [25,26]. This aligns with our findings, as 65% of the cases in our study were rural residents with a deficit in access to otolaryngology and dental care.

The Romanian pediatric HIV/AIDS cohort emerged during the late 1980s and early 1990s due to unsafe medical practices, including the reuse of needles and contaminated blood transfusions, primarily affecting children under three years of age, many from institutionalized care settings. Most infections were linked to HIV-1 subtype F1, unique to Romania.

As of 30 June 2024, Romania has recorded 28,273 HIV/AIDS cases, with 18,605 progressing to AIDS and 9668 remaining HIV-positive without AIDS. HIV/AIDS-related deaths have reached 8651, and 1263 patients have been lost to follow-up. Currently, 18,359 people are living with HIV/AIDS [27].

In the first half of 2024, 321 new HIV/AIDS cases were reported (186 HIV cases and 135 AIDS cases), and 95 AIDS-related deaths occurred. Heterosexual transmission remains dominant (61%), followed by transmission by MSM (31.15%) and injection drug use (4.67%). Among tested populations, HIV contacts had an 11.6% positivity rate; MSM, 1.68%; and prisoners, 13.64% [27].

ART coverage was estimated at 80–90%, but 42% of new cases were already at the AIDS stage, highlighting the urgent need for earlier diagnosis, prevention efforts, and adherence support [27].

The most common transmission route in the study group was sexual transmission, as with other regions from our country [27]. At the national level, heterosexual transmission remains the primary mode of HIV infection, followed by injection drug use and MSM-associated transmission [28,29].

We reflect on the changes in the stomatognathic system to raise awareness among colleagues from other medical fields and specialties. HIV/AIDS infection occurs and must be recognized in various medical domains.

The limitations of this study were caused by restricted access to other medical services, such as otolaryngology and dental care, during the COVID-19 pandemic. This reduced access may have delayed the diagnosis of HIV-related manifestations in the stomatognathic system. Consequently, early detection and interdisciplinary collaboration remain crucial for the timely management of HIV/AIDS cases. Another limitation is the lack of long-term follow-up data, which restricted our ability to conclude the progression and long-term outcomes of ENT and dental conditions in our HIV-positive patients.

Another limitation of this study lies in its retrospective design, which may be affected by the quality and completeness of the available data. Challenges such as incomplete medical records and the inability to account for all confounding factors at the time of data collection are inherent to retrospective analyses. Additionally, recall bias may impact the accuracy of self-reported symptoms or medical history.

Furthermore, the absence of a control group limits our ability to determine whether the observed outcomes—such as the high incidence of oropharyngeal manifestations—are specific to the HIV-positive population or are also prevalent in HIV-negative individuals or other demographic groups. This restricts the generalizability of this study’s findings and highlights the need for future comparative studies.

The key to the management of HIV-positive patients is a multidisciplinary approach, with the aid of different specialists (including ENT specialists and dentists) and access to antiretroviral therapy. We must find and eliminate obstacles affecting patients and the healthcare system in order to increase ART uptake [30,31,32]. Regular screening for oral and ENT manifestations can lead to earlier diagnosis of opportunistic infections and improved quality of life. Additionally, enhancing communication between primary care providers and specialists is essential for delivering comprehensive and continuous care [33].

Although oral candidiasis is a well-known and widely reported opportunistic infection in immunosuppressed patients with HIV, our findings contribute novel insight by analyzing this manifestation in a uniquely structured regional cohort from northeastern Romania. Romania remains home to one of the largest pediatric HIV populations in Europe, primarily infected with HIV-1 subtype F1—a globally rare but locally endemic variant with long-term clinical implications [9].

In contrast to most Western European datasets, our cohort included a high proportion of patients from rural areas (65%), where access to dental and ENT specialists remains limited. This disparity likely contributes to diagnostic delays and more advanced presentations at the time of hospital admission. Additionally, gender differences were noted: male patients showed a higher prevalence of co-infections and more severe ENT involvement, findings consistent with previous Eastern European studies [34].

When compared to other regional reports, such as the EuroAIDS cohort, which shows higher AIDS-related mortality in Eastern Europe, our data reinforce the need for improved early detection strategies and integrated care models tailored to local realities. These regional differences underscore the importance of continuing to document and analyze HIV-related complications in underrepresented settings.

## 5. Conclusions

This study’s findings underscore the critical role of the early diagnosis and management of oropharyngeal manifestations in improving patient outcomes. Integrating routine otorhinolaryngological assessments into the standard care for HIV-positive individuals is imperative, especially for those with CD4+ T cell counts below 200 cells/μL. Antifungal treatments, particularly fluconazole, demonstrated high efficacy, underscoring their importance in clinical management.

Additionally, we must improve access to HIV testing, with community outreach initiatives being especially valuable, and always provide counseling and fast referrals to medical facilities to start ART to support these outreach initiatives. Finally, to complete the management plan for people living with HIV/AIDS infection to support their quality of life, a complete evaluation must be conducted by both a dentist and an ENT specialist.

## Figures and Tables

**Table 1:** Study group patients with lesions of the stomatognathic system by the year of admission.

Year of Admission	Number of HIV/AIDS Infection Cases Admitted to Our Hospital (n)	Number of HIV/AIDS Infection Cases Admitted to Our Hospital with Lesions of the Stomatognathic System (n)
2020	281	3
2021	379	104
2022	774	141
2023	1223	139
2024	1239	165

**Table 2:** Distribution of new HIV/AIDS cases by age and gender.

Age (Years)	Total Patients (n, %)	Male (n)	Female (n)
18–29	59 (10.68%)	38	21
30–39	255 (46.19%)	165	90
40–49	132 (23.91%)	84	48
50–59	60 (10.86%)	36	24
Over 60	41 (7.42%)	35	6
Total	552 (100%)	358	194

**Table 3:** The route of transmission of the study group.

Route of Transmission	n	%
Sexual transmission Heterosexual MSM	331 198	59.96 36.21
Intravenous drug use	6	1.08
Perinatal	17	3.07

**Table 4:** Distribution of cases by CD4+ T-lymphocyte level and gender.

CD4+ T-Lymphocyte Level	Male		Female		Total	
	n	%	n	%	n	%
0–199 cells/μL	108	19.66	71	12.86	179	32.52
200–499 cells/μL	123	22.18	81	14.67	204	36.85
>500 cells/μL	127	23	42	7.63	169	30.63

**Table 5:** Distribution of new HIV/AIDS cases by co-infections.

Co-infections	Men		Women		Total	
	n	%	n	%	n	%
Oral Candidiasis	335	60.68	174	31.52	509	92.21
HBV	76	13.76	34	6.15	110	19.91
HCV	15	2.71	12	2.17	27	4.88
Tuberculosis	49	8.87	33	5.97	82	14.84

**Table 6:** Dental and ENT manifestations were recorded in our study.

Diagnosis	Gender	Total Patients (n, %)
Ethmoiditis	Female Male	4 cases, (0.72%) 2 cases, (0.36%)
Dental Abscesses/Rhinosinusitis	Male	5 cases, (0.90%)
Mastoiditis	Female Male	3 cases (0.54%) 3 cases (0.54%)
Nasal Septum Deviation	Female	1 case (0.18%)
Nasal Septum Deviation, Sinusitis/Rhinosinusitis	Male	2 cases (0.36%)
Otitis	Male	2 cases (0.36%)
Sinusitis/Rhinosinusitis	Female Male	9 cases (1.63%) 8 cases (1.44%)
Sinusitis/Rhinosinusitis, Temporomandibular Arthritis	Female	1 case (0.18%)
Temporomandibular Arthritis	Female	1 case (0.18%)
Oral Candidiasis	Male Female	335 cases, 60.68% 174 cases, 31.52%
Gingivitis	Male Female	100 cases, 18.2% 76 cases, 13.77%
Periodontitis	Male Female	123 cases, 22.28% 75 cases, 13.59%

**Table 7:** Regression analysis results for oral candidiasis predictors.

Variable	Coefficient (B)	*p*-Value	Odds Ratio (OR)	95% Confidence Interval (CI)
CD4+ T-Lymphocyte Count (per 100 cells/µL)	−0.30	<0.001	0.74	0.66–0.83
HIV Viral Load (log10 copies/mL)	−0.05	0.82	NS˚	—
Gender (Male vs. Female)	0.09	0.80	NS	—
ART Adherence (Good vs. Poor)	−0.22	0.55	NS	—
HBV Co-infection	−0.39	0.33	NS	—
HCV Co-infection	−0.92	0.15	NS	—
Tuberculosis Co-infection	−0.54	0.20	NS	—
Sociodemographic Characteristics (Urban vs. Rural)	−1.36	<0.001	0.26	0.14–0.47
Smoking (Smoker vs. Non-Smoker)	+1.25	<0.001	3.5	2.30–5.40
Gingivitis (Present vs. Absent)	+0.85	0.004	2.35	1.30–4.25
Periodontitis (Present vs. Absent)	+1.10	0.002	3.02	1.50–6.00

˚NS: Not Significant.

**Table 8:** *Candida* spp. diagnosis among smokers and non-smokers.

Smoking Status	Total Patients	Patients with *Candida* spp. Diagnosis	Percentage (%)
Smokers	442	265	60%
Non-Smokers	110	22	20%

**Table 9:** Treatment used for ENT manifestations and dental abscesses.

Treatment	Patients (n, %)	Number of Days
Aminopenicillin: Amoxicillin–Clavulanate	13 (2.35%)	7–10 days
Second-Generation Cephalosporin: Cefuroxime	5 (0.90%)	7 days
Third-Generation Cephalosporin: Ceftriaxone	5 (0.90%)	7 days
Third-Generation Cephalosporin: Ceftazidime	8 (1.44%)	7 days
Fluoroquinolone: Ciprofloxacin	12 (2.17%)	7–14 days
Antifungal: Fluconazole	509 (92.21%)	3 days

**Table 10:** Fluconazole treatment outcome in HIV patients with oropharyngeal candidiasis.

Parameter	n	%
Total Patients with Oropharyngeal Candidiasis	509	100
Patients Treated with Fluconazole 150 mg/oral/per day/3 days	509	100
Patients with Negative Follow-up Tongue Swab (after treatment)	407	80

**Table 11:** The antiretroviral regimen that was used.

Antiretroviral Regimen	n	%
Single-pill regimen	462	83.69
Multi-pill regimen	90	16.31

**Table 12:** Distribution of HIV/AIDS cases by ART regimen.

ART Regimen	n	%
Integrase inhibitors	309	55.97
Protease inhibitors	29	5.25
NRTIs + 2 NNRTIs	200	36.23
Other	14	2.53

## Data Availability

All data generated or analyzed during this study are included in this published article.

## Abbreviations

The following abbreviations are used in this manuscript:

ENT	Ear, neck, and throat;
OHL	Oral hairy leukoplakia;
HIV	Human immunodeficiency virus;
AIDS	Acquired immunodeficiency syndrome;
ART	Antiretroviral therapy;
WHO	World Health Organization;
NS	Not significant.
